# Hierarchical attention transformer provides assistant suggestions for orbital rejuvenation surgery

**DOI:** 10.3389/fmed.2025.1532195

**Published:** 2025-03-06

**Authors:** Xiang Lian, Xin Hu, Guannan Li, Siqi Wu, Yihao Liu, Ke Qin, Kai Liu

**Affiliations:** ^1^Department of Plastic and Reconstructive Surgery, Shanghai Ninth People’s Hospital, Shanghai Jiao Tong University School of Medicine, Shanghai, China; ^2^Shanghai Key Laboratory of Tissue Engineering, Shanghai Ninth People’s Hospital, National Tissue Engineering Center of China, Shanghai Jiao Tong University School of Medicine, Shanghai, China; ^3^Shanghai Institute for Plastic and Reconstructive Surgery, Shanghai, China; ^4^University of Electronic Science and Technology of China, Chengdu, Sichuan, China; ^5^Ecole Polytechnique, Institut Polytechnique de Paris, Palaiseau, France

**Keywords:** periocular aging, Hierarchical Attention Transformer (HATrans), AI-based decision-making, multi-label classification, lower blepharoplasty, double eyelid surgery, epicanthal fold surgery, lateral canthoplasty

## Abstract

**Background:**

Early detection of periocular aging is a common concern in cosmetic surgery. Traditional diagnostic and treatment methods often require hospital visits and consultations with plastic surgeons, which are costly and time-consuming. This study aims to develop and evaluate an AI-based decision-making system for periocular cosmetic surgery, utilizing a Hierarchical Attention Transformer (HATrans) model designed for multi-label classification in periocular conditions, allowing for home-based early aging identification.

**Methods:**

This cross-sectional study was conducted at the Department of Plastic and Reconstructive Surgery at Shanghai Jiao Tong University School of Medicine’s Ninth People’s Hospital from September 1, 2010, to April 30, 2024. The study enhanced the Vision Transformer (ViT) by adding two specialized branches: the Region Recognition Branch for foreground area identification, and the Patch Recognition Branch for refined feature representation via contrastive learning. These enhancements allowed for better handling of complex periocular images.

**Results:**

The HATrans model significantly outperformed baseline architectures such as ResNet and Swin Transformer, achieving superior accuracy, sensitivity, and specificity in identifying periocular aging. Ablation studies demonstrated the critical role of the hierarchical attention mechanism in distinguishing subtle foreground-background differences, improving the model’s performance in smartphone-based image analysis.

**Conclusion:**

The HATrans model represents a significant advancement in multi-label classification for facial aesthetics, offering a practical solution for early periocular aging detection at home. The model’s robust performance supports its potential for assisting clinical decision-making in cosmetic surgery, facilitating accessible and timely treatment recommendations.

## 1 Introduction

The appearance of youthful, vibrant, and lively eyes is often regarded as a key element of facial aesthetics. To achieve this ideal, various orbital rejuvenation procedures have been developed, both in academic research and clinical practice (1). These procedures include medial and lateral canthoplasty, as well as upper and lower blepharoplasty. Regardless of the specific surgical approach, the concept of aesthetic units is critical for ensuring cohesive treatment of the orbital region (2, 3). Conditions such as monolids and ptosis can create a tired or dull appearance, particularly in flatter facial contours (4). Narrow palpebral fissures reduce corneal visibility, and a shortened lateral canthus can disrupt facial symmetry, while an extended lateral canthus aligns more closely with aesthetic ideals (5, 6).

With growing economies and improving living standards, the desire for cosmetic enhancement has increased globally (7). Eyelid surgery is now one of the most commonly performed cosmetic procedures worldwide, underscoring the importance of the eyes in facial aesthetics. The main objective of orbital rejuvenation surgery is to restore youthful proportions to the face and emphasize the eyes (8, 9). However, there are no universally accepted standards for these procedures, and no single technique has gained widespread recognition. Surgeons typically base their recommendations on aesthetic evaluations of the periorbital area and patient preferences (10). Yet, many patients lack the necessary expertise in aesthetic evaluation, leading to uncertainty in determining the most effective treatment. Additionally, surgeons often rely on their own experience and preferences, which can limit the objectivity of initial treatment decisions (11). A model capable of offering surgical recommendations during orbital rejuvenation diagnosis would therefore optimize treatment plans and enhance post-operative monitoring (12).

Recent advances in artificial intelligence (AI) and deep learning (DL) have made automated facial feature extraction a reality (13). DL models, particularly the Vision Transformer (ViT), have demonstrated remarkable performance in computer vision tasks by learning from vast datasets of natural images (14).

Unlike traditional machine learning methods, which require manual feature extraction, DL can process raw data and autonomously develop representations for pattern recognition. Despite its success in medical image analysis, no validated DL method exists for diagnosing and recommending treatments for orbital rejuvenation.

In this study, we introduce a novel intelligent decision-making system for periocular cosmetic surgery, utilizing a Hierarchical Attention Transformer (HATrans) model specifically designed for multi-label classification in periocular surgeries (15). The model was developed using data collected from cohorts of patients at Shanghai Jiao Tong University School of Medicine’s Ninth People’s Hospital between September 1, 2010, and April 30, 2024. Our method extends the Vision Transformer (ViT) architecture by incorporating two additional branches: the Region Recognition Branch and the Patch Recognition Branch. The Region Recognition Branch focuses on identifying foreground areas related to specific attributes of the periocular region, such as the lateral canthus, while the Patch Recognition Branch refines the representations of both foreground and background features using contrastive learning (16).

This architecture addresses the complexity of multi-label classification by simultaneously predicting multiple surgical interventions required for the periocular area. Extensive experiments demonstrate that HATrans significantly outperforms baseline models such as ResNet (17) and Swin Transformer (18), achieving superior accuracy across multiple evaluation metrics, including sensitivity, specificity, and overall classification accuracy. Additionally, ablation studies confirmed the importance of the hierarchical attention mechanism in HATrans, particularly its ability to capture subtle differences between foreground and background regions that are crucial for making accurate surgical recommendations.

The HATrans model also showed strong performance in identifying periocular aging from smartphone images alone, allowing for convenient, at-home assessments of eye conditions. This capability not only provides early diagnostic potential but also offers classified treatment recommendations based on a comprehensive analysis of the patient’s periocular characteristics. The results of this study establish a new state-of-the-art benchmark for multi-label classification in medical image analysis related to facial aesthetics, paving the way for AI-driven decision-making systems to support clinical judgment in cosmetic surgeries.

## 2 Dataset and problem

### 2.1 Patient cohorts

**Figure 1:**
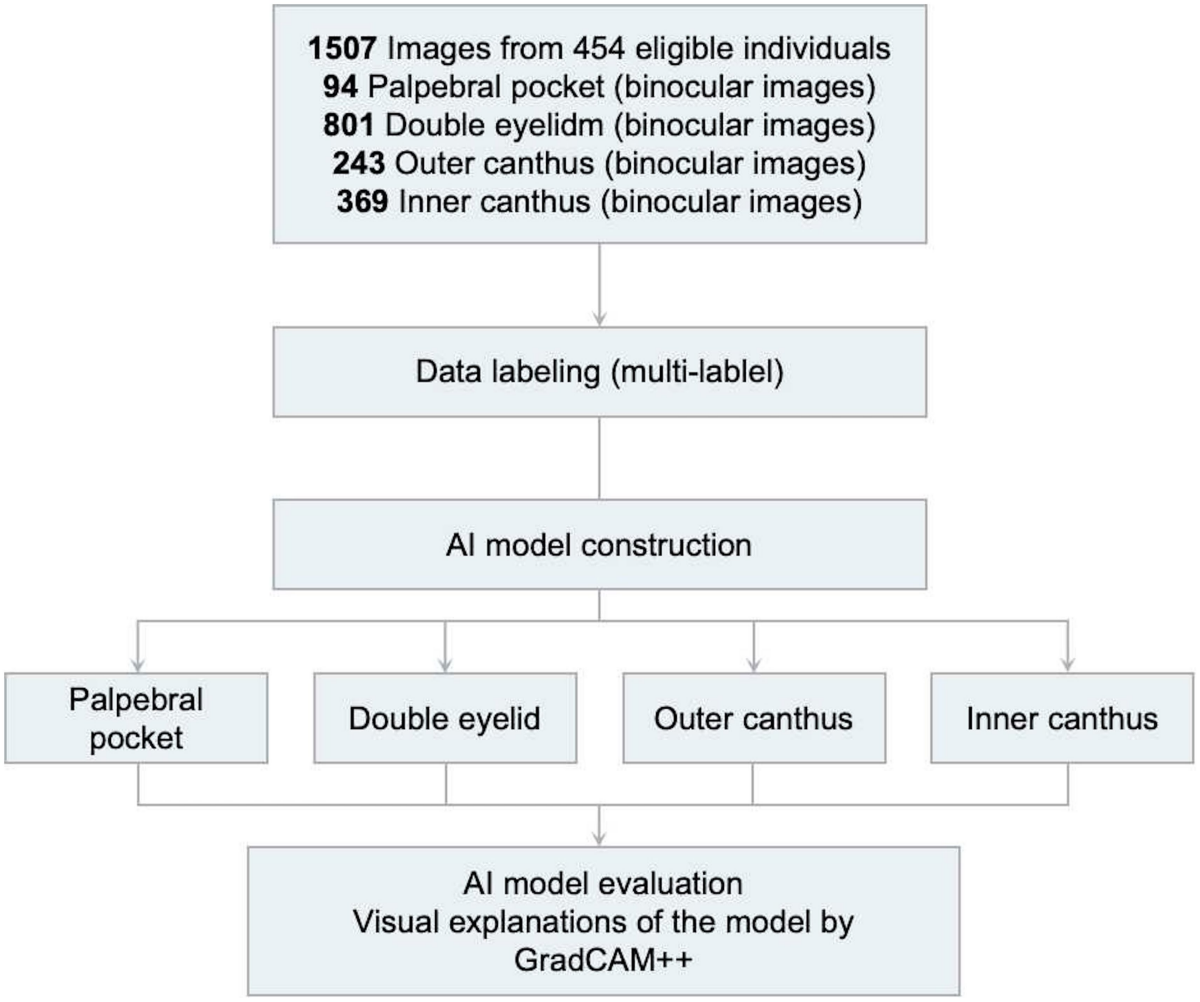
Overview of the first cohort.

We collected two independent patient cohorts. The first cohort, originating from China, was divided into a training set and a validation set, used for model selection and hyperparameter optimization (19). This cohort consisted of 454 Chinese patients from the Ninth People’s Hospital, Shanghai Jiao Tong University School of Medicine, who received consultations and treatments between June 2010 and April 2020 (Figure 1). The inclusion criteria were: patients who sought and were indicated for periocular cosmetic surgery and were completely satisfied with the results; exclusion criteria were: (a) patients with significant facial trauma; (b) those with facial deformities; (c) missing or poor-quality image data; and (d) incomplete or missing clinical follow-up data. Secondly, we gathered data for a test cohort, used solely to evaluate the final model. This cohort was composed of periocular cosmetic patients from the Ninth People’s Hospital, who received treatment between August 2003 and April 2021. The same inclusion and exclusion criteria were applied.

### 2.2 Photo acquisition

All photographs were taken using a mobile phone, capturing three angles: frontal, oblique, and lateral views. The images were taken using a smartphone from a distance of 0.5 m from the patient. The patient was instructed to remove their spectacles, maintain their head upright, and stare straight ahead. The study received ethical approval from the Ethics Committee at the Shanghai Ninth People’s Hospital affiliated to Shanghai Jiao Tong University School of Medicine (approval no. SH9H-2023-T279-1) (20). All procedures performed in the study were in accordance with the ethical standards of the institutional and national research committee, and with the 1964 Helsinki declaration and its later amendments or comparable ethical standards (21).

### 2.3 Problem description

This work addresses a multi-label classification problem in predicting required surgeries for eyes based on a labeled dataset. Unlike traditional single-label classification, where each sample belongs to one class, this task involves predicting multiple labels per sample, as an eye can require several surgeries simultaneously.

Formally, given a dataset $D=\left\{\left(x_{1},y_{1}\right),\ldots ,\left(x_{N},y_{N}\right)\right\}$ , where each $x_{i}\in {\mathbb{R}}^{d}$ represents the feature vector of an eye, and $y_{i}=\left[y_{i,1},y_{i,2},\ldots ,y_{i,k}\right]\in {\left\{0,1\right\}}^{k}$ is a binary label vector indicating the required surgeries out of k possible types, the objective is to learn a mapping function $f:{\mathbb{R}}^{d}\to {\left\{0,1\right\}}^{k}$ such that:


$${\hat{y}}_{i}=f\left(x_{i}\right)\;for\;i=1,\ldots ,N\text{,}$$

where ${\hat{y\;}}_{i}$ represents the predicted label vector. The key challenge is accurately predicting multiple labels while considering interdependencies among surgery types.

### 2.4 Evaluation metric

We evaluate model performance using subset accuracy, a strict metric commonly applied in multi-label classification. Subset accuracy measures the proportion of samples for which the predicted label vector exactly matches the ground truth across all k labels. It is defined as:


$$\mathrm{Accuracy}=\frac{1}{N}\overset{N}{\underset{i=1}{\sum }}I\left({\hat{y}}_{i}=y_{i}\right)\text{,}$$

where $I\left(\cdot \right)$ is an indicator function that returns 1 if the predicted label vector ${\hat{y\;}}_{i}$ matches the ground truth $y_{i}$ , and 0 otherwise.

## 3 Methods

**Figure 2:**
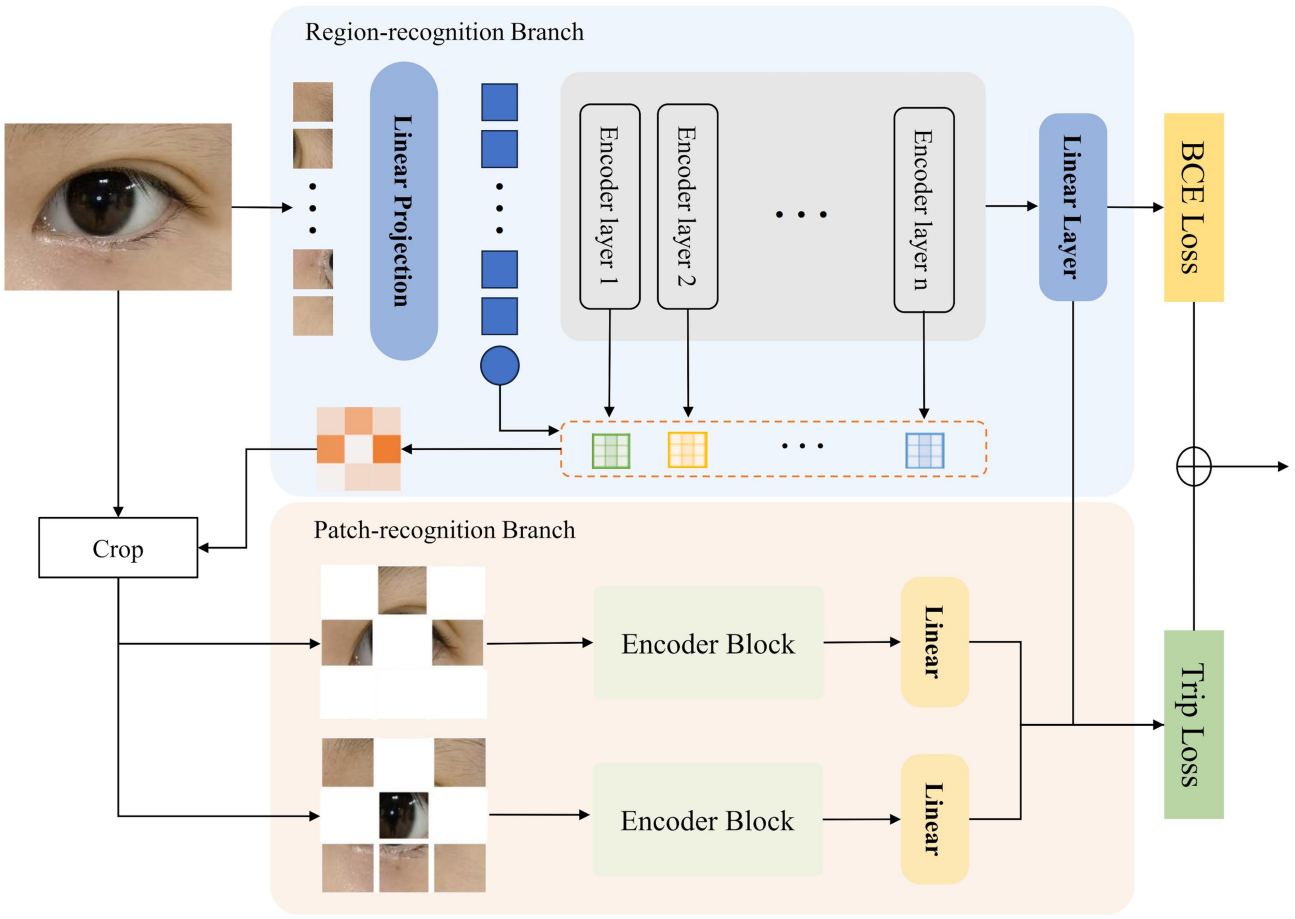
The architecture of the proposed Hierarchical Attention Transformer (HATrans). The model includes a Region-recognition branch for identifying attribute-relevant regions and a Patch-recognition branch that refines foreground and background features, using Binary Cross-Entropy and Triplet loss for optimization.

Figure 2 illustrates the structure of our proposed Hierarchical Attention Transformer (HATrans), which enhances the basic ViT model by introducing two additional decoder branches. The primary Region-recognition branch focuses on identifying attribute-relevant foreground regions and separating them from background areas. The two additional Patch-recognition branches explore finer-grained attribute contexts within the foreground regions and learn attribute-specific foreground-background representations through contrastive learning. The architecture of HATrans is detailed in the following subsections.

### 3.1 Region-recognition branch

The Vision Transformer (ViT) adopts the Transformer architecture for image recognition by viewing an image as a sequence of patches, transforming image processing into a sequence modeling task. Given an input image $x\in {\mathbb{R}}^{H\times W\times C}$ , ViT splits the image into N patches of size P×P . Each patch is flattened into a vector $z_{p}\in {\mathbb{R}}^{N\times \left(P^{2}\cdot C\right)}$ . Learnable position embeddings $E_{pos}\in {\mathbb{R}}^{\left(N+1\right)\times D}$ are added to retain positional information. A class token $z_{\mathrm{class}}\in {\mathbb{R}}^{D}$ is prepended to the sequence, which is used for classification:


$$z_{0}=\left[z_{\mathrm{class}};z_{0}^{1}+E_{pos}^{1};\ldots ;z_{0}^{N}+E_{pos}^{N}\right]$$

The resulting sequence $z_{0}$ is fed into the Transformer encoder, which consists of alternating layers of multi-head self-attention (MHSA) and multi-layer perceptrons (MLPs). Each Transformer encoder layer applies LayerNorm (LN), followed by MHSA and MLP layers:


$$z\prime_{L}=\mathrm{MHSA}\left(LN\left(z_{L-1}\right)\right)+z_{L-1}$$

$$z_{L}=MLP\left(LN\left(z\prime_{L}\right)\right)+z\prime_{L}$$

where L denotes the layer index.

The Region-recognition branch extends the ViT framework by integrating a mechanism to distinguish attribute-relevant foreground regions from background areas. Inspired by TransFG, this branch leverages the attention weights from each encoder layer to guide region separation.

After the patch embeddings are processed through the Transformer encoder, we aggregate the multi-head self-attention (MHSA) weights across all layers using the Hadamard product, enabling relevant attention features to accumulate and gradually enhance through layers. Formally, the accumulated attention map for each patch i after L layers is defined as:


$$A_{m}={⊙}_{l=0}^{L-1}A_{l}$$

where ⊙ denotes the Hadamard product applied across all layers’ attention maps $A_{l}$ .

After obtaining the accumulated attention map $A_{m}$ , we focus on its diagonal elements, which represent the self-attention scores of each token, reflecting the importance of each token relative to itself and other tokens. Let g be the vector of diagonal elements, defined as:


$$g=\mathrm{diag}\left(A_{m}\right)=\left[g_{0},g_{1},g_{2},\ldots ,g_{i},\ldots ,g_{N}\right]$$

Each $g_{i}$ represents the importance score of the i -th token. We apply a threshold τ to g to determine the foreground and background regions. Tokens with importance scores above τ are classified as foreground, while those below τ are classified as background:


$$M_{F}=\{\begin{array}{ll} 1, & \mathrm{if}\;g_{i}>\tau \\ 0, & \mathrm{otherwise} \end{array}$$

The background mask is defined as $M_{B}=1-M_{F}$ . By leveraging the diagonal elements of the accumulated attention map, this method effectively captures the tokens most critical for identifying discriminative regions, enabling the model to focus on important areas for fine-grained recognition tasks.

### 3.2 Patch-recognition branch

The Patch-recognition branch in HATrans is designed to further refine the feature representations by focusing separately on the foreground and background regions identified in the Region-recognition branch. This branch consists of two sub-branches: a foreground sub-branch and a background sub-branch. Both sub-branches share the same transformer architecture as the main Region-recognition branch, ensuring consistent feature extraction while adapting to the specific context of each region.

The foreground sub-branch shares parameters with the transformer layers in the Region-recognition branch, allowing the learned attention and feature representations to be directly leveraged. This shared parameter strategy maintains consistency across different stages of feature extraction while reducing the overall model complexity.

Formally, let $z_{F}$ and $z_{B}$ represent the patch tokens from the identified foreground and background regions, respectively. Both $z_{F}$ and $z_{B}$ are processed through their respective transformer structures:


$$z_{F}^{l}=\mathrm{Transformer}\left(z_{F}^{l-1}\right),\;z_{B}^{l}=\mathrm{Transformer}\left(z_{B}^{l-1}\right)$$

where l denotes the layer index. The foreground sub-branch uses shared parameters with the main Region-recognition branch, while the background sub-branch has independent parameters, allowing it to focus specifically on the unique characteristics of background regions.

This separation and specialization of the two sub-branches enhance the model’s ability to capture subtle distinctions between foreground and background features. The refined feature representations are later integrated for final classification, providing more robust attribute-specific predictions.

### 3.3 Multi-scale branch training objectives

The training objectives for HATrans involve a combination of Binary Cross-Entropy (BCE) loss and Triplet loss. These losses jointly optimize global classification and the distinction between foreground and background features across the multi-scale branches.

The BCE loss is applied to the output of the main Region-recognition branch. Specifically, the class token’s final feature is used for classification. Given the predicted logits $y_{p}$ from the linear classifier and the ground truth labels $y_{t}$ for a batch of size B , the BCE loss is defined as:


$$L_{B}=-\frac{1}{B}\overset{B}{\underset{i=1}{\sum }}\left[y_{t}^{\left(i\right)}\cdot \log\left(y_{p}^{\left(i\right)}\right)+\left(1-y_{t}^{\left(i\right)}\right)\cdot \log\left(1-y_{p}^{\left(i\right)}\right)\right]$$

To enhance the discriminative power between the learned foreground and background features, we introduce a Triplet loss. The anchor, positive, and negative samples are derived from the features extracted by the three different branches: the Region-recognition branch, the foreground Patch-recognition branch, and the background Patch-recognition branch. Specifically, $z_{R}$ , $z_{F}$ , and $z_{B}$ represent the feature embeddings from these branches, respectively. The Triplet loss is calculated as follows:


$$L_{T}=\frac{1}{B}\overset{B}{\underset{i=1}{\sum }}\max\left(sim\left(z_{R}^{\left(i\right)},z_{F}^{\left(i\right)}\right)-sim\left(z_{R}^{\left(i\right)},z_{B}^{\left(i\right)}\right)+\alpha ,0\right)$$

where $sim\left(\cdot ,\cdot \right)$ denotes the cosine similarity function, and α is a margin parameter.

The final training objective combines the BCE loss and the Triplet loss as:


$$L=\lambda_{B}L_{B}+\lambda_{T}L_{T}$$

where $\lambda_{B}$ and $\lambda_{T}$ control the relative importance of each loss term. This combination allows the model to jointly optimize global classification and region-specific distinctions, leading to improved fine-grained recognition performance.

## 4 Experiments and discussion

In this section, we present experiments conducted to evaluate the proposed Hierarchical Attention Transformer (HATrans) model, exploring its performance in the given multi-classification task.

### 4.1 Experimental setting

#### 4.1.1 Dataset

We utilized our proposed eye dataset, which includes four types of recommended surgical procedures: eye bag removal, double eyelid surgery (blepharoplasty), medial canthoplasty (inner canthus correction), and lateral canthoplasty (outer canthus correction). The dataset consists of a total of 1,507 images collected from 454 patients who met the research criteria: 94 images for identifying eye bags, 801 images for recognizing ptosis, single eyelids, and upper eyelid skin laxity, 243 images for identifying short palpebral fissures and overly elevated lateral canthi, and 369 images for recognizing epicanthal folds. The dataset is divided into a training set and a test set, with about 20% of the images allocated to the test set: 18 images for identifying eye bags, 162 images for recognizing ptosis, single eyelids, and upper eyelid skin laxity, 48 images for identifying short palpebral fissures and overly elevated lateral canthi, and 75 images for recognizing epicanthal folds. Each image is annotated with multiple labels to indicate the relevant surgical procedures, allowing us to address this task as a multi-label classification problem. The dataset is balanced to ensure diverse representation across different surgical types, and the images have been preprocessed to normalize the input for model training.

#### 4.1.2 Implementation details

Our proposed model is implemented in three variants—Base, Large, and Huge—each corresponding to the pre-trained Vision Transformer (ViT) models. The Base, Large, and Huge variants are initialized with the pre-trained weights from ViT-Base, ViT-Large, and ViT-Huge, respectively, allowing us to leverage the transfer learning capabilities of the original models.

The input resolution for all models is set to 224 × 224 pixels. During training, we use the AdamW optimizer with a learning rate of $10^{-4}$ , a weight decay of 0.05, and a momentum parameter of 0.9. The learning rate follows a cosine annealing schedule, with a linear warm-up phase of 10 epochs. The total number of training epochs is set to 100, with the learning rate reduced after 60 and 80 epochs. To ensure a consistent evaluation, a batch size of 16 is employed for all training processes. Data augmentation techniques, such as random cropping, horizontal flipping, and color jitter are used to enhance the model’s generalization.

In our experiments, we also compare our model against several widely adopted baselines, including ResNet-50, ResNet-101, EfficientNet, Swin Transformer, and DeiT. Since these baseline models are primarily designed for binary classification tasks, we adapted them to the multi-label classification scenario by employing a Binary Cross Entropy Loss function. This modification ensures a fair comparison and allows evaluation of their effectiveness in the context of predicting multiple required eye surgeries.

### 4.2 Quantitative analysis

Table 1 presents a comparison of our proposed Hierarchical Attention Transformer (HATrans) model against several baseline architectures, including ResNet-50, ResNet-101, EfficientNet, Swin Transformer, Vision Transformer (ViT), and DeiT, across multiple evaluation metrics. Our model, in all configurations—Base, Large, and Huge—outperforms the baseline models, demonstrating the effectiveness of the proposed hierarchical attention mechanism for the multi-label eye surgery classification task.

**Table 1:** Ablation study results for different components of the HATrans model.

Variant	ACC	Rec	F1	AUC
Baseline ViT (without region-recognition or patch-recognition)	0.7632	0.7485	0.7510	0.8250
+ Region-recognition branch	0.8145	0.8022	0.8080	0.8715
+ Patch-recognition branches (without contrastive learning)	0.8273	0.8150	0.8182	0.8820
+ Contrastive learning in patch-recognition branches	0.8602	0.8575	0.8550	0.9087

Each row shows the performance after adding a specific component, highlighting the impact of Region-recognition, Patch-recognition branches, and contrastive learning.

The ResNet models show comparatively lower performance, reflecting the limitations of purely convolutional architectures in capturing complex dependencies across image patches (22). Transformer-based models, such as ViT and DeiT (23), exhibit a significant improvement due to their self-attention mechanisms, which are better suited for learning relationships among diverse image features (24, 25).

The HATrans model achieves the best performance, with the Huge variant showing substantial gains across key metrics. This performance improvement can be attributed to the hierarchical attention mechanism, which enhances the basic ViT model by incorporating the Region-recognition and Patch-recognition branches. The Region-recognition branch allows for effective separation of attribute-relevant regions, while the Patch-recognition branches refine the feature representation through contrastive learning between foreground and background areas. These enhancements enable HATrans to capture subtle attribute-specific contexts more effectively, resulting in improved classification capabilities compared to the baseline models.

### 4.3 Ablation studies

To evaluate the contributions of the different components within our proposed Hierarchical Attention Transformer (HATrans) model, we conducted a series of ablation experiments, as summarized in Table 1. Specifically, we aim to understand the impact of each major architectural addition, including the Region-recognition branch, the Patch-recognition branches, and the use of contrastive learning between foreground and background features.

#### 4.3.1 Effect of region-recognition branch

The Region-recognition branch plays a critical role in distinguishing attribute-relevant foreground regions from the background. To assess its impact, we compare the performance of the full model with a variant where the Region-recognition branch is removed, effectively making the model a standard Vision Transformer without the capability to separate foreground from background regions. The results show a noticeable decline in accuracy and F1-score, indicating that explicitly modeling foreground-background separation allows the model to focus on more informative regions, which is essential for fine-grained classification.

#### 4.3.2 Effect of patch-recognition branches

To evaluate the benefit of the Patch-recognition branches, we conducted experiments by removing these branches while keeping the Region-recognition branch intact. The resulting model only distinguishes between foreground and background but does not refine attribute-specific representations. The absence of Patch-recognition branches led to a reduced performance across all metrics, highlighting the importance of further exploring finer-grained attribute contexts through separate foreground and background learning.

#### 4.3.3 Effect of contrastive learning

The Patch-recognition branches employ contrastive learning to enhance the distinction between foreground and background features. We performed an ablation where contrastive learning was replaced with a standard classification loss applied separately to each sub-branch. The results indicate that the contrastive learning objective significantly improves model performance, particularly in distinguishing subtle variations between the foreground and background features. This suggests that explicitly contrasting the two regions helps in learning discriminative features, enhancing the overall model’s capability to identify distinct attributes.

#### 4.3.4 Combined impact

Finally, we analyzed the combined impact of removing both the Region-recognition and Patch-recognition branches. This resulted in a substantial drop in performance, approaching that of the baseline Vision Transformer. These results validate that both branches play complementary roles, with the Region-recognition branch providing essential spatial context and the Patch-recognition branches enhancing feature discrimination through multi-scale learning and contrastive objectives.

### 4.4 Quality analysis

To further assess the effectiveness of our proposed Hierarchical Attention Transformer (HATrans) model, we conducted a qualitative analysis by visualizing the attention maps generated by the final layer of the model. Specifically, we visualized the attention weights from the Region-recognition branch to highlight the attribute-relevant regions that the model focuses on during prediction (26). We compared these attention maps with those generated by ResNet, Vision Transformer (ViT), and our HATrans model.

**Figure 3:**
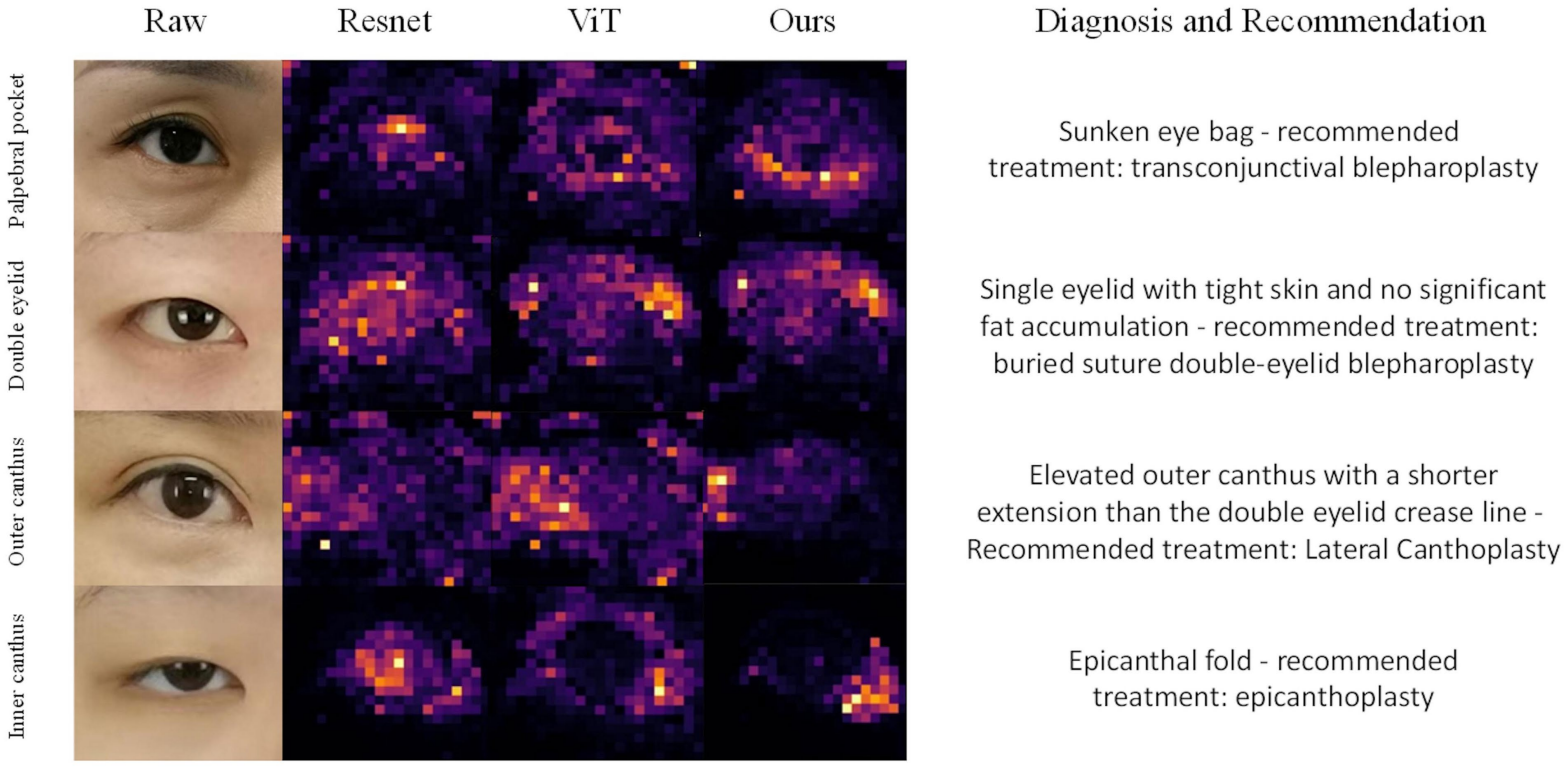
Grad-CAM visualizes the attention mechanisms of ResNet, Vision Transformer (ViT), and our proposed model when focusing on different regions of the eye, specifically the eyelid bags, double eyelid, outer canthus and inner canthus.

Figure 3 presents the attention maps produced by these models on representative samples from the eye surgery dataset. The attention maps from HATrans demonstrate a more focused and well-defined separation of the foreground regions, effectively highlighting the areas most relevant for predicting the required surgeries. In contrast, ResNet, which relies on convolutional feature extraction, shows less distinct attention and often fails to capture specific regions of interest accurately. The Vision Transformer produces more coherent attention maps compared to ResNet, but the attention is still diffused across irrelevant background regions. Our HATrans model, by leveraging the Region-recognition and Patch-recognition branches, achieves superior localization, allowing it to concentrate on the most critical features while excluding unnecessary background information. This targeted focus results in more accurate predictions, illustrating the advantages of our hierarchical attention mechanism over traditional convolutional networks and baseline transformer models.

The qualitative results demonstrate that our hierarchical approach allows for a more targeted focus on discriminative features. The Region-recognition branch enables the model to effectively differentiate between significant foreground areas and irrelevant background, leading to sharper and more interpretable attention maps. Additionally, the Patch-recognition branches refine these attribute-specific regions through contrastive learning, further enhancing the model’s ability to discern subtle distinctions. As a result, the HATrans model exhibits superior localization capabilities compared to other state-of-the-art transformer-based models, thereby achieving higher accuracy in multi-label periocular surgery classification.

## 5 Results

### 5.1 Data characteristics

Among the 454 patients meeting the research criteria, a total of 1,507 images were collected to construct the model: 94 images for identifying eye bags, 801 for recognizing ptosis, monolids, and eyelid laxity, 243 for identifying short palpebral fissures and excessive upward tilt of the lateral canthus, and 369 for detecting epicanthal folds (Figure 1).

The specific patient counts were as follows: 31 patients with infraorbital hollowing, ptosis, or herniation; 241 patients with ptosis, monolids, or upper eyelid laxity; 73 patients with short palpebral fissures or excessive upward tilt of the lateral canthus; and 109 patients with epicanthal folds.

Moreover, some patients presented with comorbidities. For example, 23 patients (5%) underwent simultaneous eye bag and upper eyelid surgery, 41 patients (9%) underwent both epicanthal and lateral canthal surgeries, 74 patients (16%) underwent epicanthal and upper eyelid procedures, and 52 patients (11%) had upper eyelid and lateral canthal surgeries.

### 5.2 Model performance

Sensitivity, specificity, and accuracy of the model are presented, as shown in Table 2. The model exhibited comparable performance in recognizing periocular aging and providing recommendations for both male and female subjects.

**Table 2:** Model performance in predicting palpebral pocket, double eyelid, outer canthus and inner canthus.

Performance variant	Sensitivity	Specificity	Accuracy	AUC	NLR	NPV	PLR	PPV	F1-score
Palpebral pocket	0.8113	0.7789	0.7143	0.8190	0.2115	0.8766	0.289	0.8178	0.7871
Double eyelid	0.8001	0.8156	0.8956	0.8588	0.1917	0.8956	0.2728	0.7901	0.8723
Outer canthus	0.7917	0.8312	0.8467	0.8328	0.1978	0.8798	0.531	0.8276	0.8564
Inner canthus	0.8267	0.8577	0.8535	0.8413	0.2018	0.8465	0.412	0.8158	0.8322

### 5.3 Model interpretation via heatmaps

We employed GradCAM++ to assess the influence of different regions in the periocular area on the AI model’s classification outcomes. The heatmaps, generated from network weights combined with feature maps, illustrated the importance of individual pixels in image classification. Warmer colors in the heatmap indicate areas of higher significance. As depicted in Figure 3, the model assigns varying weights to different periocular regions when identifying the four types of periocular deformities.

### 5.4 Permission to reuse and copyright

Permission must be obtained for use of copyrighted material from other sources (including the web). Please note that it is compulsory to follow figure instructions.

### 5.5 Surgical descriptions

#### 5.5.1 Lower blepharoplasty

Lower blepharoplasty, commonly known as eye bag surgery, is a procedure designed to correct signs of aging around the lower eyelids, such as skin laxity, herniation of orbital fat, and hypertrophy of the orbicularis oculi muscle. The surgery involves precise separation of skin and muscle, repositioning of orbital fat, and removal of excess skin and muscle tissue to restore the lower eyelid’s anatomical structure and rejuvenate the periocular contour (27).

**Figure 4:**
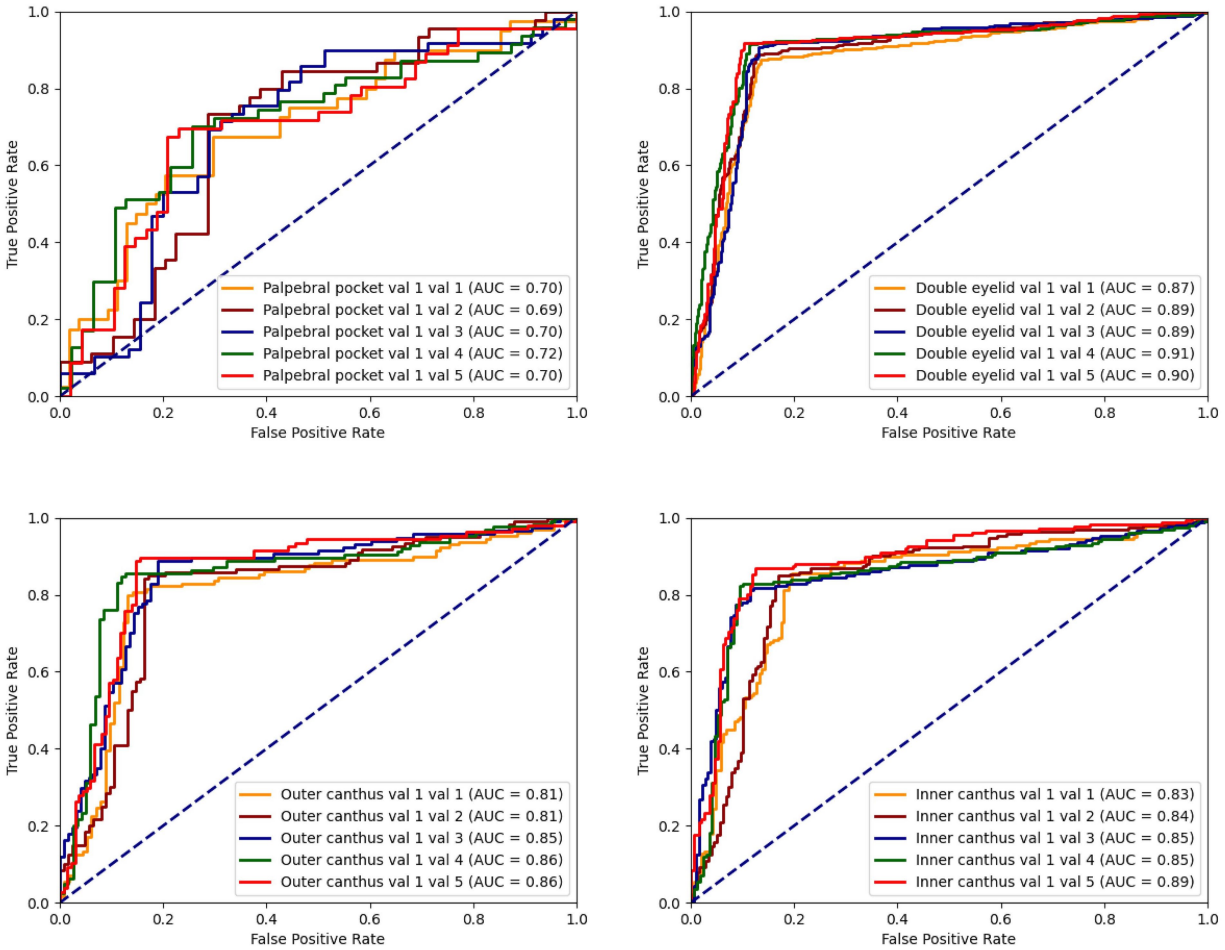
The ROC curve for the classification model concerning palpebral pocket, double eyelid, outer canthus, and inner canthus illustrates the situation between the true positive rate (TPR) and the false positive rate (FPR).

The model’s accuracy in identifying patients requiring eye bag surgery is 71.43%. In practical applications, the recommendations provided by the model were accepted in 92% of cases. Early intervention with eye bag surgery can effectively eliminate eye bags, restore a youthful appearance, reduce skin laxity, and lower the difficulty of surgery, thereby accelerating recovery and mitigating long-term skin damage caused by eye bags (Figure 4).

#### 5.5.2 Double eyelid surgery

In East Asia, double eyelid surgery is a popular cosmetic procedure, with a wide audience across genders. According to statistical data, female patients constitute the majority of double eyelid surgery recipients, accounting for approximately 80–90%, while male patients represent 10–20%. This gender disparity reflects women’s greater focus on periocular aesthetics. However, this ratio may vary across regions, cultures, and over time. Notably, the proportion of male patients undergoing double eyelid surgery is rising, reflecting evolving societal views and increased acceptance of cosmetic procedures among men.

Beyond creating the double eyelid appearance, the procedure plays a significant role in periocular rejuvenation. With age, skin laxity and fat accumulation contribute to periocular aging.

**Figure 5:**
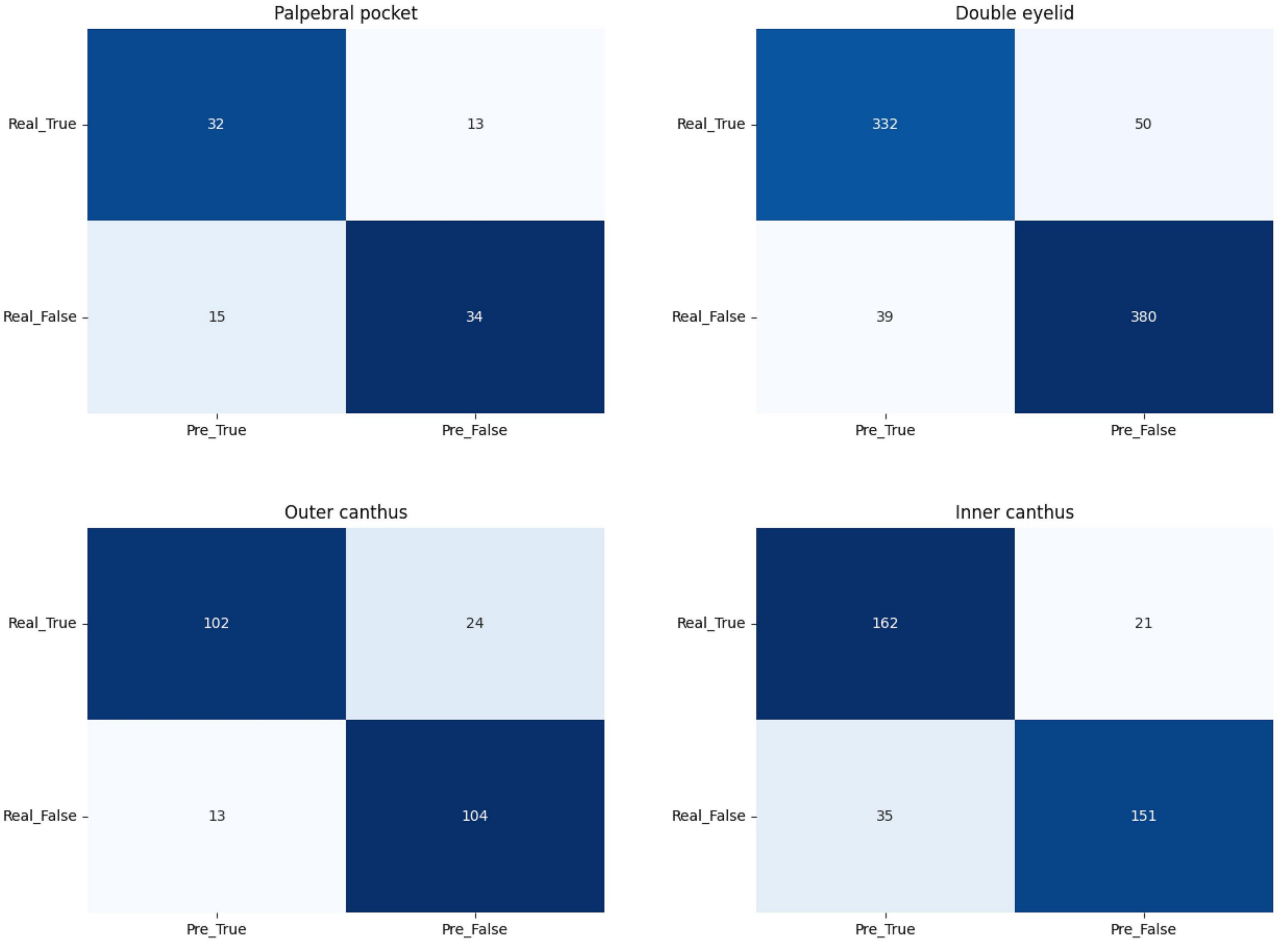
The confusion matrix for palpebral pocket, double eyelid, outer canthus, and inner canthus.

The model’s accuracy in identifying ptosis, monolids, and upper eyelid laxity is 89.56%. In practical applications, 89.5% of the recommendations were accepted. Early double eyelid surgery can leverage the skin and tissue’s elasticity to promote faster recovery, reduce the risk of complications, and yield more natural and lasting aesthetic outcomes, boosting patients’ confidence and quality of life (Figure 5).

#### 5.5.3 Double eyelid surgery

Epicanthoplasty is a precise surgical procedure aimed at correcting epicanthal folds and improving the length and shape of the palpebral fissure. Studies indicate that the prevalence of epicanthal folds ranges from 50 to 90% in East Asian populations, significantly affecting the aesthetic appearance of the eyes. The surgery typically involves making a 1.5–2.0 cm micro-incision at the epicanthus, through which approximately 1.0–1.5 mm of skin and muscle tissue is removed. The medial canthal ligament is then released and repositioned. Depending on the patient’s condition, epicanthoplasty can increase the palpebral fissure length by 2–5 mm, visibly enhancing the horizontal width of the eyes and achieving more balanced proportions according to aesthetic standards.

Postoperative evaluations reveal a patient satisfaction rate of 85–95%, with a low complication rate (infection, bleeding, or scarring risk less than 5%). The model’s accuracy in identifying epicanthal folds is 85.35, and 94% of the recommendations were adopted in clinical practice.

#### 5.5.4 Lateral canthoplasty

Lateral Canthoplasty is a procedure designed to extend the horizontal width of the palpebral fissure. The surgery involves severing part of the lateral canthal ligament and fixing it in a new position, coupled with excising an appropriate amount of skin and muscle tissue from the outer eyelid. According to clinical data, the procedure can increase the palpebral fissure width by 3–6 mm, significantly enhancing the aesthetic appeal of the eye shape.

The model’s accuracy in identifying lateral canthal deformities is 84.67, and 94% of its recommendations were accepted. Clinical observations suggest that appropriate lateral canthoplasty may correct certain cases of strabismus, although the exact mechanism requires further investigation.

## 6 Discussion

To our knowledge, this study represents the first instance of using patient facial photographs to simultaneously identify infraorbital hollowing, ptosis, monolids, short palpebral fissures, and epicanthal folds. The use of smartphone-based applications to detect periocular aging and recommend four common periocular rejuvenation surgeries alleviates some of the burdens on healthcare systems. However, this study has limitations. First, as a single-center, cross-sectional study with a small sample size, further multi-center investigations are necessary to improve the algorithm’s generalizability. Additionally, due to insufficient recording of patient baseline characteristics (such as age, occupation, skin type, and exercise habits), the algorithm’s functionality is constrained. Collecting more comprehensive patient information may enhance the model’s performance. Moreover, the uneven distribution of cases among the four conditions may explain the model’s lower sensitivity in detecting eye bags (28, 29). Increasing the number of images of patients with infraorbital conditions could improve the model’s performance.

## 7 Conclusion

This study demonstrates that AI-based detection models exhibit strong performance in accurately identifying periocular aging from smartphone images. These results indicate that such models can assist individuals in identifying infraorbital hollowing, ptosis, monolids, short palpebral fissures, and epicanthal folds. Some types of periocular aging can potentially lead to complications such as trichiasis, corneal, and conjunctival irritation, or vision problems. In some cases, they may also induce forehead wrinkles or headaches due to compensatory mechanisms such as excessive eyebrow raising (30). Early identification and intervention can prevent these issues from worsening, optimizing patient experience by reducing delays in diagnosis and treatment. This pre-diagnostic tool can thus play a critical role in timely medical decision-making, saving patients time and improving overall outcomes.

Furthermore, by facilitating the early detection of periocular aging, the model can contribute to a more equitable distribution of limited medical resources. Individuals with significant periocular aging that impacts facial aesthetics may benefit from specific algorithmic assessments that detect early signs of aging. Based on historical datasets, the model is designed to provide treatment recommendations under simple and practical conditions, with the aim of maximizing overall aesthetic improvement through a single surgical procedure.

The model can also serve as an auxiliary diagnostic tool for physicians in primary healthcare settings (31). As the dataset continues to expand, it is expected that the accuracy and personalization of the model’s recommendations will improve, thus better serving clinical diagnosis and patient care (32).

Overall, the improvements made in this study in addressing multi-label classification issues within the domain of medical image analysis for facial aesthetics establish a new high standard (33). The development of this model not only enhances current technologies but also suggests its potential for wide application in supporting decision-making in clinical plastic surgery. Specifically, the findings from this study are expected to provide scientific and precise decision support for a variety of cosmetic surgeries, promoting the advancement and refinement of plastic and cosmetic surgery practices.

This completes the enhanced and formalized conclusion section, reinforcing the academic rigor and clinical relevance of the study. The refined structure and content ensure clarity in presenting the model’s clinical implications while highlighting its potential future development.

## Data Availability

The raw data supporting the conclusions of this article will be made available by the authors, without undue reservation.
